# Household transmission of Streptococcus pneumoniae, Alberta, Canada.

**DOI:** 10.3201/eid0501.990120

**Published:** 1999

**Authors:** J D Kellner, A P Gibb, J Zhang, H R Rabin

**Affiliations:** Foothills Medical Centre, Calgary, Alberta, Canada. jim.kellner@crha-health.ab.ca


### Household Transmission of *Streptococcus pneumoniae*, Alberta, Canada

**James D. Kellner,*† A. Patrick Gibb,†‡**
**Jenny Zhang,§ and Harvey R. Rabin*†**
*Foothills Medical Centre and Alberta Children's Hospital, Calgary, Alberta, Canada; †University of Calgary, Alberta, Canada; ‡Calgary Laboratory Services, Alberta, Canada; and §Provincial Laboratory of Public Health, Calgary, Alberta, Canada

Proven or presumptive multidrug-resistant *Streptococcus pneumoniae* pneumonia was diagnosed simultaneously in three married couples in Alberta, Canada. The pair of isolates from each couple had identical antibiotic resistance profiles, serotypes, and pulsed-field gel electrophoresis patterns. One or more of these cases could have been prevented by *S. pneumoniae* vaccine.

Outbreaks of *Streptococcus pneumoniae* (antibiotic resistant and nonresistant) have been reported from child-care centers, nursing homes, hospitals, military camps, homeless shelters, and penal institutions (1-6). Simultaneous cases within households have rarely been reported (7-11); such cases require common exposure and transmission, as well as similar likelihood of disease in the hosts or increased virulence in the pathogen.

In December 1996 and January 1997, three married couples with multidrug-resistant *S. pneumoniae* (MDRSP) were admitted to Foothills Medical Centre in Calgary. The couples were not admitted on the same day. None of the couples lived with children, although couple C had daily contact with children. All patients received appropriate antibiotic therapy after their culture and antibiotic sensitivity results were known. We reviewed each patient's health record (Table) and were able to contact two of the three couples for further information.

*S. pneumoniae* were identified by standard methods. MICs were determined by E-Test (AB Biodisk, Solna, Sweden) and classified as susceptible (S), intermediate resistant (I), or fully resistant (R) to each antibiotic, according to National Committee for Clinical Laboratory Standards guidelines (12). Serotyping of *S. pneumoniae* was performed by the Quellung reaction technique at the National Centre for Streptococcus, Edmonton. Electrophoretic fingerprinting of *S. pneumoniae* was performed by pulsed-field gel electrophoresis (PFGE) of DNA digested with *Sma*1 (BRL, Gaithersburg, MD). The PFGE patterns were classified as indistinguishable, related, or different according to criteria suggested by Tenover (13).

The diagnosis of *S. pneumoniae* pneumonia in couple A was confirmed by positive blood cultures, chest X-ray lobar pneumonia, and disease-compatible clinical findings. Patient 1 in couple A was a health records clerk at Foothills Medical Centre. Her illness was complicated soon after admission by empyema, which was drained; the fluid was *S. pneumoniae*-negative. Vertebral osteomyelitis was suspected from clinical evidence 18 days after admission and was confirmed by bone scan; no diagnostic culture was obtained. Osteomyelitis in this patient was presumably caused by *S. pneumoniae*. The initial 7-day course of cefuroxime (to which *S. pneumoniae* was resistant) may not have cleared the infection and thus allowed secondary seeding to bone.

Address for correspondence: James D. Kellner, Division of Infectious Diseases, Alberta Children's Hospital, 1820 Richmond Road, SW, Calgary, Alberta T2T 5C7, Canada; fax: 403-229-7665; e-mail: jim.kellner@crha-health.ab.ca.

Table. Clinical and laboratory features of three couples with *Streptococcus pneumoniae* pneumonia

| | Couple A | | Couple B | | Couple C | |
|---|---|---|---|---|---|---|
| Feature | Patient 1 | Patient 2 | Patient 1 | Patient 2 | Patient 1 | Patient 2 |
| Age (yrs) | 62 | 61 | 72 | 71 | 39 | 37 |
| Chronic conditions | Hypertension, diabetes | Gout, 3 previous MIs[a] | Hypertension, CAD[b] | COPD[c] | Recurrent sinusitis | Recurrent sinusitis |
| Smoker | No | No | Yes | Yes | Yes | Yes |
| *S. pneumoniae* vaccine | No | No | Unknown | Unknown | No | No |
| Recent antibiotics | None | None | Unknown | Unknown | >3 courses in previous year | >3 courses in previous year |
| Others in home | None | None | None | None | None | None |
| Initial complaints | URTI[d] symptoms, cough, fever | URTI[d] symptoms, cough, fever | URTI[d] symptoms, cough, fever, chest pain | URTI[d] symptoms, cough, fever, chest pain, eye discharge | Burn, recent URTI[d] symptoms, cough, fever | Burn, recent URTI[d] symptoms, cough, fever |
| Physical exam | Febrile, ↑HR[e], ↑RR[f], severe distress, ↓breath sounds | Febrile, ↑HR[e], ↑RR[f], ↓breath sounds | Febrile, ↑HR[e], ↑RR[f], ↓breath sounds, ↓$O_2$ saturation | Febrile, ↑HR[e], ↑RR[f], ↓breath sounds | Febrile, ↑distress on ventilator, ↓breath sounds, crepitations | Febrile, ↑distress on ventilator, ↓breath sounds, crepitations |
| Chest X-ray (admission or as noted) | Right upper lobe consolidation | Right lower lobe consolidation | Bibasilar consolidation | Extensive right-sided consolidation | Day 3 – extensive bilateral consolidation | Day 2 – extensive bilateral consolidation |
| Admitting diagnosis | Right lobe pneumonia | Bilateral pneumonia | Pneumonia | Lobar pneumonia | Burn | Burn |
| Discharge diagnosis | Right upper lobe pneumonia | Right lower lobe pneumonia | Pneumonia | Lobar pneumonia | Burn, complicated by pneumonia | Burn, complicated by pneumonia fatal sepsis |
| Complications | Empyema, osteomyelitis | None | None | None | None | Died |
| Source of isolate | Day 1 - blood | Day 1 - blood | Day 1 - sputum (4+[i]) | Day 1 - sputum (3+[i]) | Day 3 - ETT[g] (4+[i]) | Day 2 - BAL[h] ($10^5$ CFU/mL[i]) |
| Gram stain | Not applicable | Not applicable | GPC resembling *S. pneumoniae*[j] | GPC resembling *S. pneumoniae*[j], GNB[k] | GPC resembling *S. pneumoniae*[j] | GPC resembling *S. pneumoniae*[j] |
| Other potential pathogens when pneumonia diagnosed | None | None | None | *H. influenzae* (3+[i]) | GNB[k] | *H. influenzae* ($10^3$ CFU/mL[i]) |
| Antibiotic susceptibility[l] | | | | | | |
| Penicillin | 2 R | 1.5 I | 1.5 R | 2 I | 1.5 I | 1 I |
| Cefuroxime | 4 R | 6 R | 3 R | 4 R | 6 R | 4 R |
| Ceftriaxone | 1 I | 0.5 S | 0.75 S | 0.38 S | 0.75 S | 0.75 S |
| TMP/SMX[m] | ≥32 R | ≥32 R | ≥32 R | ≥32 R | ≥32 R | ≥32 R |
| Erythromycin | 0.25 S | 0.25 S | 16 R | 16 R | 0.25 S | 0.25 S |
| Serotype | 14 | 14 | 9V | 9V | 9V | 9V |
| PFGE pattern[n] | AA | AA | BB | BB | BC | BC |

[a]Myocardial infarction.
[b]Coronary artery disease.
[c]Chronic obstructive pulmonary disease.
[d]Upper respiratory tract infection.
[e]Heart rate.
[f]Respiratory rate.
[g]Endotracheal tube.
[h]Bronchoalveolar lavage.
[i]For sputum or ETT aspirates, 3+ & 4+ reflect growth on the third and fourth set of streaks, respectively, on the culture plate; for BAL, sample fluid is an approximately 100-fold dilution of lung fluid.
[j]Gram-positive lancet-shaped cocci found singly, in pairs or in short chains.
[k]Gram-negative coccobacilli.
[l]Antibiotic susceptibilities reported as MIC (micrograms/mL) and as S (susceptible), I (intermediate) or R (resistant) (NCCLS criteria).
[m]TMP/SMX (trimethoprim/sulfamethoxazole).
[n]Pulsed-field gel electrophoresis.

Couple B (who could not be reached for further information) had had recent visitors from Texas (one a hospital worker) with upper respiratory tract infections. *S. pneumoniae* pneumonia was presumptively diagnosed in this couple on the basis of symptoms, signs, and chest X-rays compatible with the diagnosis of pneumonia, as well as sputum samples, which had gram-positive lancet-shaped cocci identified on Gram stain and grew *S. pneumoniae*. From the sputum of patient 2 in couple B, gram-negative bacilli were identified on Gram stain; *Haemophilus influenzae* was also isolated. Thus, this patient may have been coinfected, or primarily infected, with *H. influenzae*. The patient's blood cultures were negative; a blood culture was not performed on patient 1 in couple B.

Couple C was admitted with severe burns and inhalation injuries after the stove in their two-room trailer exploded. They had had recurrent sinusitis and other respiratory infections in the previous year since moving to their trailer, which had poor air circulation. Patient 1 of this couple was taking antibiotics at the time of admission, and patient 2 had recently completed a course of antibiotics. The diagnosis of pneumonia (patient 1 on day 3 of admission and patient 2 on day 2) was made on the basis of recent upper respiratory symptoms and fever, diminished breath sounds, crepitations, and disease-compatible chest X-ray findings (previous films had been normal), which made pneumonia more likely than noninfectious conditions such as acute lung syndrome. The presumptive diagnosis of *S. pneumoniae* as the etiologic agent in the case of patient 1, couple C, was made on the basis of the initial endotracheal tube aspirate, which had gram-positive lancet-shaped cocci identified on Gram stain and grew *S. pneumoniae*. Only gram-positive lancet-shaped cocci were identified from the initial bronchoalveolar lavage of patient 2 on Gram stain, and *S. pneumoniae* grew in much greater numbers than *H. influenzae*. Blood cultures, performed for couple C only after antibiotic therapy was started, were negative. Patient 2 died of septic shock 20 days after admission, with *Candida albicans* in his blood. The bronchopneumonia never resolved clinically, although *S. pneumoniae* was not isolated from any further cultures. Thus, *S. pneumoniae* may have been a contributing factor to, but not likely the direct cause, of this patient's death.

The identical susceptibility patterns, serotypes, and PFGE patterns indicate that both partners in each couple were infected with the same multidrug-resistant *S. pneumoniae* strain. Couples A and B apparently had community-acquired pneumonia. Although couple C contracted pneumonia 48 to 72 hours after admission, each partner entered the hospital already infected with MDRSP; the infecting organisms were identical, and no other recognized cases of nosocomial MDRSP occurred at Foothills Medical Centre at the time of their admission (they were admitted 1 month before couple B, who were also infected with serotype 9V MDRSP). Couple A may have been exposed to MDRSP as a result of one partner's work in a tertiary-care hospital; couple B as a result of one partner's exposure to a health-care worker with respiratory symptoms. At the time of these cases, the prevalence of penicillin-nonsusceptible *S. pneumoniae* infections in Calgary was approximately 10% (A.P. Gibb, unpub. data).

None of these patients had received *S. pneumoniae* vaccine, yet each had one or more risk factors for infection (advanced age, exposure to young children, smoking, and chronic lung or heart disease). Couple C had a history of recent antibiotic use, the predominant risk factor for antibiotic-resistant infections.

In Canada, the *S. pneumoniae* vaccine is recommended for all persons ≥65 years old and persons ≥2 years with identified risk factors (14). Despite the vaccine's reasonable effectiveness, its use has been very low in Canada until recently (fewer than 12 doses per 10,000 population distributed annually [15,16]). The vaccine has been provided free of charge to persons with medical indications, but not to healthy persons 65 years of age and older and not as part of a routine vaccination schedule (17). Some provinces (including Alberta, beginning in 1998) have begun to routinely provide the vaccine to all persons at risk. The current incidence of invasive *S. pneumoniae* infections in Calgary is 20 per 100,000 per year overall and 87 per 100,000 per year in those older than 64 years of age (J.D. Klein, unpub. data).

Outbreaks of *S. pneumoniae* disease occur in institutions with crowding, poor air quality, or increased host susceptibility (2,4,6). These factors may also exist within households (9,11). Couple C, for example, lived in a very crowded space with poor air circulation.

The rate at which secondary *S. pneumoniae* infections occur in household contacts of index patients with invasive disease is not known, but rare cases have been reported (7-11). Factors contributing to secondary infections include the likelihood of nasopharyngeal infection due to exposure to the index patient or a common source, susceptibility to the strain of the index infection, and likelihood that colonization will lead to disease rather than to development of asymptomatic immunity. Data on contemporaneous nasopharyngeal carriage of the outbreak strain by household contacts are limited. A recent study from Gambia found carriage in 8.5% of household contacts, compared with 21% in an older U.S. study (18,19). In healthy adults, the prevalence of circulating *S. pneumoniae* antibodies is low (4% to 34%, depending on the serotype); however, two thirds of adults have protective antibody within 1 month of colonization (20). Approximately 15% of children who acquire a new *S. pneumoniae* strain nasopharyngeally in a nonoutbreak setting acquire clinical disease (usually otitis media); this rate is unknown for adults (21). In contrast, during a recent nursing-home pneumonia outbreak, 23% of residents were infected with the *S. pneumoniae* outbreak strain, and 4% became ill (22). The median age of residents was 85 years; only 4% had received *S. pneumoniae* vaccine.

Increased use of *S. pneumoniae* vaccine may prevent MDRSP pneumonia within households and among persons living in crowded conditions.

### Acknowledgments

We thank Sheila Robertson for performing the chart reviews, James Talbot and Marguerite Lovgren for serotyping, and Kevin Fonseca for directing the pulsed-field gel electrophoresis.

Dr. Kellner is an assistant professor of Pediatrics and Microbiology and Infectious Diseases at the University of Calgary, Canada. His research interests include *S. pneumoniae* infections and antimicrobial resistance.

### References

1. Cherian T, Steinhoff MC, Harrison LH, Rohn D, McDougal LK, Dick J. A cluster of invasive pneumococcal disease in young children in day care. JAMA 1994;271:695-7.
2. Hoge CW, Reichler MR, Dominguez EA, Bremer JC, Mastro TD, Hendricks KA, et al. An epidemic of pneumococcal disease in an overcrowded, inadequately ventilated jail. N Engl J Med 1994;331:643-8.
3. Quick RE, Hoge CW, Hamilton DJ, Whitney CJ, Borges M, Kobayashi JM. Underutilization of pneumococcal vaccine in nursing homes in Washington State: report of a serotype-specific outbreak and a survey. American Journal of Medicine 1993;94:149-52.
4. Mercat A, Nguyen J, Dautzenberg B. An outbreak of pneumococcal pneumonia in two men's shelters. Chest 1991;99:147-51.
5. Musher D, Groover J, Reichler M, Riedo F, Schwartz B, Watson D, et al. Emergence of antibody to capsular polysaccharides of *Streptococcus pneumoniae* during outbreaks of pneumonia: association with nasopharyngeal colonization. Clin Infect Dis 1997;24:441-6.
6. Mandigers CMPW, Diepersloot RJA, Dessens M, Mol SJM, van Klingeren B. A hospital outbreak of penicillin-resistant pneumococci in the Netherlands. Eur Respir J 1994;7:1635-9.
7. Asmar BI, Dajani A. Concurrent pneumococcal disease in two siblings. Am J Dis Child 1982;136:946-7.
8. Fenton PA, Spencer RC, Savill JS, Grover S. Pneumococcal bacteremia in mother and son. Brit Med J 1983;287:529-30.
9. Collingham KE, Littlejohns PD, Wiggins J. Pneumococcal meningitis in a husband and wife. J Infect 1985;10:256-8.
10. Tilghman RC, Finland M. Pneumococcic infections in families. J Clin Invest 1936;15:493-9.
11. Heffron R. Pneumonia: with special reference to pneumococcus lobar pneumonia. Cambridge: Harvard University Press; 1939.
12. National Committee for Clinical Laboratory Standards. Table 2G. MIC Interpretive Standards (μg/mL) for *Streptococcus pneumoniae*. Villanova (PA): National Committee for Clinical Laboratory Standards; 1998. p. 68-9.
13. Tenover FC, Arbeit RD, Goering RV, Mickelsen PA, Murray BE, Persing DH, et al. Interpreting chromosomal DNA restriction patterns produced by pulsed-field gel electrophoresis: criteria for bacterial strain typing. J Clin Microbiol 1995;33:2233-9.
14. National Advisory Committee on Immunization. Canadian Immunization Guide. 4th ed. Ottawa: Health and Welfare Canada, 1993.
15. Fedson DS. Influenza and pneumococcal vaccination in Canada and the United States, 1980-1993: what can the two countries learn from each other? Clin Infect Dis 1995;20:1371-6.
16. Fedson DS. Pneumococcal vaccination in the United States and 20 other developed countries, 1981-1996. Clin Infect Dis 1998;26:117-23.
17. Epidemiology CDCA. Alberta immunization manual. Edmonton: Alberta Health; 1996.
18. Lloyd-Evans N, O'Dempsey TJ, Baldeth I, Secka O, Demba E, Todd JE, et al. Nasopharyngeal carriage of pneumococci in Gambian children and their families. Pediatr Infect Dis J 1996;15:866-71.

19. Smillie WG, Jewett OF. The relationship of immediate family contact to the transmission of type-specific pneumococci. American Journal of Hygiene 1940;32:79-88.
20. Musher DM, Groover JE, Rowland JM, Watson DA, Struewing JB, Baughn RE, et al. Antibody to polysaccharides of *Streptococcus pneumoniae*: prevalence, persistence and response to revaccination. Clin Infect Dis 1993;17:66-73.
21. Gray BM, Converse III GM, Dillon HC. Epidemiologic studies of *Streptococcus pneumoniae* in infants: acquisition, carriage, and infection during the first 24 months of life. J Infect Dis 1980;142:923-33.
22. Nuorti JP, Butler JC, Crutcher JM, Guevera R, Welch D, Holder P, et al. An outbreak of multidrug-resistant pneumococcal pneumonia and bacteremia among unvaccinated nursing home residents. N Engl J Med 1998;338:1861-8.

